# Descemet membrane endothelial keratoplasty: analysis of clinical outcomes of patients with 8–10 years follow-up

**DOI:** 10.1007/s10792-021-02176-3

**Published:** 2022-01-08

**Authors:** Julia M. Weller, Friedrich E. Kruse, Theofilos Tourtas

**Affiliations:** Department of Ophthalmology, Friedrich-Alexander University Erlangen-Nürnberg, Schwabachanlage 6, 91054 Erlangen, Germany

**Keywords:** Fuchs’ endothelial corneal dystrophy, Descemet Membrane Endothelial Keratoplasty (DMEK)

## Abstract

**Purpose:**

This study aimed to evaluate the clinical outcomes up to 10 years after Descemet membrane endothelial keratoplasty (DMEK).

**Methods:**

In this retrospective, consecutive, single-center case series the medical files of eyes which have received DMEK between 2009 and 2012 for the treatment of endothelial dysfunction was evaluated regarding follow-up time and clinical outcomes.

Annual examinations of best-corrected visual acuity (BCVA), endothelial cell density (ECD), central corneal thickness (CCT) of 66 eyes which fulfilled the criterion of a minimum of 8 years follow-up were analyzed.

**Results:**

BCVA improved from 0.55 ± 0.37 logMAR (*n* = 54) to 0.15 ± 0.11 (*n* = 47) in eyes without ocular comorbidities one year after DMEK (*p* < 0.001), and remained stable up to 10 years after DMEK. Mean ECD decreased to 744 ± 207 cells/mm^2^ (*n* = 39) after 9 years, and to 729 ± 167 cells/mm^2^ (*n* = 21) after 10 years, respectively. CCT decreased from 650 ± 67 μm before DMEK to 525 ± 40 μm (*n* = 56) after 1 year, increasing slowly to 563 ± 40 µm (*n* = 39) after 9 years, and to 570 ± 42 µm (*n* = 21) after 10 years, respectively. Graft failure occurred in 4 of 66 eyes after year 8. These 4 eyes required repeat DMEK after 101–127 months.

**Conclusion:**

This study shows the long-term outcomes in a small subset of DMEK grafts. Visual acuity remained stable in spite of slowly increasing corneal thickness and diminishing endothelial cell density during the 10-year period after DMEK.

**Supplementary Information:**

The online version contains supplementary material available at 10.1007/s10792-021-02176-3.

## Introduction

Descemet membrane endothelial keratoplasty (DMEK) has become a favorite method to treat endothelial dysfunction. The advantages of the method are rapid visual rehabilitation, near-normal anatomical structure of the cornea, and a low rate of graft rejections. [1–9].

The surgical technique encountered resistance by many corneal surgeons in the first years after introduction due to difficulties during the learning curve and the initially non-standardized procedure [10]. An argument against DMEK, which has been expressed in the past, is the challenging preparation of the vulnerable, thin graft with potential high endothelial cell loss during preparation and surgery diminishing survival of the graft. Much effort has been made to lower the rate of complications and thereby increase the reproducibility of the method, for example by reducing the rate of graft detachments. [11–13].

By this means, DMEK has gained popularity and has become more widespread in the US and Europe [14, 15]. Fuchs’ endothelial corneal dystrophy is the most common indication for keratoplasty worldwide, and the number of DMEK surgeries has been doubling every year between 2011 and 2014 in the United States, although Descemet’s stripping automated endothelial keratoplasty (DSAEK) is still very popular [14–16]. In Germany, DMEK has surpassed DSAEK as most common keratoplasty technique already in 2012. [15].

However, in contrast to the century-long experience gained in penetrating keratoplasty (PK), DMEK is a relatively new technique with few long-term data. The purpose of this study was to evaluate if the success of DMEK shown for the first years after surgery persists beyond the time frame of 5 years using a standardized surgical technique. [17–20].

## Methods

### Patients

In this retrospective, single-center cohort study, the long-term results after DMEK were evaluated. The main inclusion criterion was a minimum postoperative follow-up interval of 8 years.

The medical files of all DMEK surgeries performed between July 2009 and June 2012 (*n* = 450) were analyzed regarding the follow-up time. The follow-up time of all eyes undergoing surgery during this interval was obtained and the graft survival rate was calculated (data shown as supplemental Table 1 and supplemental Fig. 1). 66 eyes fulfilled the inclusion criterion of a follow-up of at least 8 years. Participation in all intermediate follow-up examinations was not required for patients to be included.

The surgeries had been performed between July 2009 and June 2012 at the Department of Ophthalmology, Friedrich-Alexander-University Erlangen-Nürnberg (FAU), Erlangen, Germany. The male/female ratio was 47%/53%, the mean age at time of surgery was 63 ± 9 years. Indication for DMEK was Fuchs endothelial corneal dystrophy in 60 eyes (91%), DMEK after failed DSAEK in 4 eyes (6%), and DMEK after failed DMEK in 2 eyes (3%). Mean follow-up time was 108 ± 11 months. 39/66 eyes (59%) fulfilled a follow-up of 9 years, 21/66 eyes (32%) a follow-up of 10 years.

The Institutional Review Board (IRB)/ Ethics Committee of the Friedrich-Alexander University Erlangen-Nürnberg approved the study (Approval ID: 64_15 Bc). The study was in adherence to the tenets of the Declaration of Helsinki. Informed consent to surgery was obtained from all patients prior to surgery.

### Corneal grafts

The donor corneoscleral tissues were obtained from eye banks in the United States (hypothermic storage at 4 °C in Optisol-GS, *n* = 20, 30%), and from Europe (via *German Society for Tissue Transplantation *(*DGFG*); organ-culture at 34 °C in Dulbecco’s modifies Eagle medium, *n* = 46, 70%). Mean donor age was 69 ± 13 years, mean death-to preservation time was 9 ± 5 h, and mean culture storage duration was 344 ± 172 h.

### Surgical technique

All surgeries were performed under general anesthesia by 3 different surgeons. On the day before DMEK, two Nd:YAG-laser iridotomies were performed to prevent postoperative pupillary block.

The surgical technique as described by Kruse et al. was used for graft preparation and transplantation [18–20]. Graft preparation was performed on the day of surgery by the surgeons themselves. Standard graft size was 8.0 mm. Triple DMEK defined as DMEK combined with cataract surgery was performed in 28 cases (42%) [1]. We used a spherical single-piece acrylic intraocular lens (46 S AcriSmart; Carl Zeiss Meditec, Jena, Germany) for implantation. [1, 3]

A repeat air injection into the anterior chamber was performed in case of clinically significant graft detachment during the early postoperative period. Graft detachment was considered significant when there was a gap of more than one full corneal thickness over more than one quadrant of the transplant. This so-called rebubbling procedure was necessary in 45% (*n* = 30) of eyes (one rebubbling: *n* = 20; two rebubblings: *n* = 5; three rebubblings: *n* = 5). The mean interval between DMEK surgery and last rebubbling was 10 ± 7 days (median 7 days, range 3–35 days).

### Postoperative medication

The postoperative standard treatment regimen consisted of topical pilocarpine 1% four times a day until the air bubble in the anterior chamber had been resorbed completely, topical ofloxacin 0.3% twice a day for 10 days, hyperosmolar eye drops 5 times a day, and topical prednisolone acetate 1% five times a day. Prednisolone eye drops were tapered monthly over 5 months and continued once a day during the first postoperative year.

### Statistics and measurements

The main outcome parameters of this study were best-corrected distance visual acuity (BCVA) in logarithm of the minimum angle of resolution (logMAR) units, endothelial cell density (ECD) in cells/mm^2^, central corneal thickness (CCT) in µm, and frequency of graft failures after the 8-years follow-up. BCVA was measured by routine visual acuity tests with number of optotypes using optimal spectacle correction.

Follow-up examinations were performed at 1 and 3 months after surgery, then annually up to 10 years after DMEK. Only the annual examinations were analyzed in this study.

CCT values were measured using Scheimpflug imaging (pachymetry at corneal apex, *Pentacam; Oculus, Wetzlar, Germany*); only scans with approval of high quality by the device were taken into account.

Endothelial cell density was analyzed using specular microscopy by two different manufacturers: from 2009 until September 2016, the device *SeaEagle* (HAI Laboratories, Lexington, MA, USA) was applied. *Tomey specular microscope EM-4000* (Tomey GmbH Technology and vision, Nürnberg, Germany) was used from October 2016 on. The automatic cell border analysis provided by the devices was used for endothelial cell density calculation. All measurements and cell border alignments were checked by an independent examiner and corrected manually in case of misalignment.

In case of low quality of the CCT or ECD measurement, the values were not evaluated in this study. Due to the retrospective design of this study, a repeat measurement was not possible afterward.

The program SPSS for Windows (version 24, SPSS Inc, Chicago, IL, USA) was used for statistical analysis of the data. We compared measurements using Wilcoxon signed-rank test with a significance level set at 5% (*P* = 0.05). Normal distribution of the data was examined with the Kolmogorov–Smirnov test showing no normal distribution.

## Results

### Visual acuity

Mean preoperative best-corrected visual acuity (BVCA) ± SD was 0.63 ± 0.43 logMAR improving to 0.19 ± 0.14 after 1 year (*P* < 0.001). Afterward, visual acuity remained stable up to 10 years after DMEK except for a slight decrease of BCVA after 8 years. BCVA (logMAR) was 0.19 ± 0.21 at year 8 (n = 54), 0.18 ± 0.20 at year 9 (n = 39), and 0.13 ± 0.18 at year 10 (*n* = 21) (Table 1). A visual acuity of 20/40 or better was achieved in 87% of eyes after 8 years, 85% after 9 years, and 91% after 10 years, respectively. 45% of eyes had a BCVA of 20/25 or better after 8 years, 51% after 9 years, 67% after 10 years, respectively.

**Table 1:** Best corrected visual acuity after Descemet membrane endothelial keratoplasty

	Baseline	1 y	2 y	3 y	4 y	5 y	6 y	7 y	8 y	9 y	10 y
BCVA including all eyes											
Mean ± SD (logMAR)	0.63 ± 0.43	0.19 ± 0.14	0.17 ± 0.16	0.17 ± 0.15	0.16 ± 0.15	0.15 ± 0.18	0.14 ± 0.18	0.15 ± 0.16	0.19 ± 0.21	0.18 ± 0.20	0.13 ± 0.18
Median	0.52	0.20	0.10	0.10	0.10	0.10	0.10	0.10	0.20	0.10	0.10
*n*	66	56	56	51	42	40	41	41	54	39	21
*P* value	–	** < 0.001**	0.694	0.828	0.866	0.396	0.216	0.319	**0.019**	0.529	0.092
BCVA excluding eyes with comorbidities											
Mean ± SD (logMAR)	0.55 ± 0.37	0.15 ± 0.11	0.12 ± 0.09	0.13 ± 0.10	0.11 ± 0.09	0.09 ± 0.11	0.10 ± 0.11	0.10 ± 0.10	0.13 ± 0.11	0.12 ± 0.12	0.10 ± 0.14
Median	0.40	0.16	0.10	0.10	0.10	0.10	0.10	0.10	0.10	0.10	0.10
*n*	55	47	45	43	35	32	37	34	45	30	15
*P* value	–	** < 0.001**	0.220	0.832	0.641	0.375	0.577	0.912	**0.013**	0.724	0.173

Best-corrected visual acuity before and at follow-up examinations up to 10 years after Descemet membrane endothelial keratoplasty, with and without exclusion of eyes with comorbidities limiting visual outcome. Wilcoxon signed-rank test was used for comparisons of the mean value with the results at the previous follow-up visit

(BCVA = best-corrected visual acuity; SD = standard deviation; logMAR = logarithm of the minimum angle of resolution, y = years)

The significance level was set at 0.05. Significant differences were marked in bold

Eleven eyes included in this study had ocular comorbidities influencing the visual potential. Ocular comorbidities comprised amblyopia (*n* = 3), epithelial basement membrane dystrophy with irregular astigmatism (*n* = 3), optic nerve atrophy (*n* = 2), macular pucker (*n* = 1), diabetic retinopathy (*n* = 1), and repeat hemorrhages into the anterior chamber due to a systemic blood coagulation disorder (*n* = 1).

Eyes without vision-impairing comorbidities (*n* = 55) had a preoperative BCVA ± SD (logMAR) of 0.55 ± 0.37 improving to 0.15 ± 0.11 (*P* < 0.001) one year after DMEK (Table 1). After the first postoperative year, the mean visual acuity values remained stable up to 10 years after DMEK (*P* > 0.05), except for a slight decrease of BCVA after 8 years. Mean BCVA (logMAR) accounted for 0.13 ± 0.11 (*n* = 45), 0.12 ± 0.12 (*n* = 30) and 0.10 ± 0.14 (*n* = 15) after 8, 9 and 10 years, respectively. In the group of eyes without comorbidities, a visual acuity of 20/40 or better was reached in 98% of eyes 8 years, in 97% 9 years, and in 95% 10 years postoperatively. 53% of eyes had a BCVA of 20/25 or better after 8 years, 61% after 9 years, and 74% after 10 years, respectively.

59% of eyes (*n* = 39) attended the 9-year visit, 32% (*n* = 21) the 10-year visit. In order to avoid a bias by comparing the mean values of different numbers of eyes attending the annual visits, we added an analysis of BCVA/ECD/CCT of the same eyes (*n* = 21) which have completed the 10-year visit (Supplemental Table 2).

### Endothelial cell density

Mean donor ECD ± SD was 2582 ± 212 cells/mm^2^ and decreased to 1504 ± 275 cells/mm^2^ after the first postoperative year (*P* < 0.001) (table 2). The cell density remained stable up to five years but decreased considerably afterward: ECD was 739 ± 197 cells/mm^2^ (*n* = 45), 744 ± 207 cells/mm^2^ (*n* = 39), and 729 ± 167 cells/mm^2^ (*n* = 21) at the 8-, 9-, and 10-year visit, respectively. Thereby, endothelial cell loss compared to the baseline measurement accounted for 71, 71, and 72%, after 8, 9, and 10 years, respectively. The decline of ECD between years 5 and 6, and between year 6 and 7 was statistically significant (*P* < 0.001).

### Central corneal thickness

Mean CCT decreased from 650 ± 67 µm at baseline to 525 ± 40 µm at the 1-year follow-up visit (− 19%, P < 0.001). A slight increase of mean CCT to 532 ± 45 µm after 2 years (*P* = 0.001) was noticed (Table 2). CCT values increased significantly between year 3 and 4 and between 4 and 5, and remained stable at an average of 559 ± 48 µm (n = 54), 563 ± 40 µm (n = 39), and 570 ± 42 µm (*n* = 21), after 8, 9, and 10 years, respectively. The CCT value before DMEK surgery (CCT at baseline) of eyes in which DMEK had been performed for the indication of failed DSAEK (*n* = 4) was excluded from the analysis since the cornea thickness is increased in these eyes at the preoperative measurement because of the DSAEK graft.

**Table 2:** Endothelial cell density and central corneal thickness after Descemet membrane endothelial keratoplasty

	Baseline	1 month	3 months	1 y	2 y	3 y	4 y	5 y	6 y	7 y	8 y	9 y	10 y
A) Endothelial cell density													
Mean ± SD (cells/mm^2^)	2582 ± 212	1486 ± 258	1456 ± 292	1504 ± 275	1384 ± 234	1377 ± 286	1389 ± 299	1373 ± 312	1107 ± 339	759 ± 202	739 ± 197	744 ± 207	729 ± 167
Median	2510	1471	1431	1494	1382	1419	1384	1413	1195	696	691	687	694
Loss in %	–	42%	43%	42%	46%	47%	46%	47%	57%	71%	71%	71%	72%
*n*	66	44	58	52	52	47	36	35	35	32	45	39	21
*P* value	–	** < 0.001**	0.368	0.116	0.061	0.786	0.496	0.136	** < 0.001**	**0.001**	0.683	0.407	0.826

	Baseline	1 y	2 y	3 y	4 y	5 y	6 y	7 y	8 y	9 y	10 y
B) Central corneal thickness											
Mean ± SD (μm)	650 ± 67	525 ± 40	532 ± 45	540 ± 44	544 ± 43	556 ± 41	553 ± 39	551 ± 41	559 ± 48	563 ± 40	570 ± 42
Median	647	531	530	539	546	567	552	548	562	565	563
*n*	66	56	53	50	42	41	40	39	54	39	21
*P* value	–	** < 0.001**	**0.001**	0.10	**0.006**	**0.04**	0.51	0.784	0.275	0.473	0.698

The significance level was set at 0.05. Significant differences were marked in bold

Endothelial cell density and central corneal thickness before and at follow-up examinations up to 10 years after Descemet membrane endothelial keratoplasty. Wilcoxon signed-rank test was used for comparisons of the mean value with the results at the previous follow-up visit

(ECD = endothelial cell density; CCT = central corneal thickness; SD = standard deviation, y = years)

### Complications

Cystoid macular edema was detected in 6 eyes (9%) during the first postoperative year by optical coherence tomography. Immunologic graft rejection or other complications as infectious keratitis, which have been described after DMEK, did not occur in any patient. Steroid-induced postoperative glaucoma occurred in 6 eyes (9%).

Four eyes of the 66 eyes included in this long-term study suffered from a graft failure after the 8-year visit. They underwent repeat DMEK after 101, 104, 114, and 127 months, respectively. There were no signs of graft rejection; all graft failures occurred due to late endothelial failure.

## Discussion

Most studies concerning the outcomes of DMEK surgery describe the results in the first 6–24 months postoperatively [3, 5, 21–23]. The aim of this study was the evaluation of the 10-year success of DMEK surgery. In our investigation of the 5-year outcomes, we had found stable visual acuity and ECD values, and a graft survival rate of 95% [17]. The 5-year results after DMEK of 500 eyes have been published recently by the Melles group: Visual acuity improved up to 36 months and remained stable afterward [24]. Endothelial cells decreased by approximately 7% per year after the first year and accounted for 55% at 5 years.

Comparative analyses of DMEK with the conventional keratoplasty methods for the treatment of FECD exist also only for the early and intermediate postoperative phase up to five years [3, 25, 26]. Woo et al. compared the 5-year outcomes of DMEK, DSAEK, and PK for the indication FECD and pseudophakic bullous keratopathy [26]. Graft survival was best in the DMEK group (97.4%), even though the endothelial cell loss after 1 year was highest in the DMEK group (39.9%). Price et al. did not find a significant difference in graft survival and ECD after DMEK compared to DSAEK at 5 years. [27] Up to now, there is no long-term (10 years) data comparing the outcomes of DMEK with DSAEK or PK.

In the analysis of the so-called midterm results, the outcomes of 250 eyes which underwent DMEK with a follow-up time up to 7 years were analyzed by Ham et al. [28] The cumulative graft survival rate was excellent (96%) and visual acuity remained stable up to 4–7 years. ECD decreased slowly with an annual decline by 9% after the first 6 months.

The longest cohort study after DMEK has been published by the Melles group recently [29]. They described a similar endothelial cell loss (− 68%) in 57 eyes out of the first 100 DMEK patients, excellent BCVA results, and a graft survival probability of 0.79 at ten years.

The highest loss of endothelial cells occurs perioperatively, most likely attributable to the graft preparation and the transplantation procedure itself. After this early cell loss, which amounts to 42% after the first postoperative month, the ECD decreased by further 30% compared to baseline in the following 9–10 years in the present study [17]. The observed cell loss of 29% between the 1st and 10th year after surgery would represent an annual cell loss of 3%. If we use this data to extrapolate further endothelial cell loss, the critical borderline for corneal compensation, which is usually assigned at values about 500 cells/mm^2^, might be crossed 12–13 years after DMEK leading to endothelial decompensation and graft failure [30]. However, Baydoun et al. have shown that 5% of corneas which were clear 7 years after DMEK had an ECD of less than 500 cells/mm^2^. [31] Therefore, extrapolation of endothelial cell loss and prediction of graft failure remains difficult.

Interestingly, the endothelial cell loss at 10 years we detected after DMEK (72%) is comparable to the 10-year data obtained for PK (76%) observed in the *Cornea Donor Study* [32]*.* The authors of the *Cornea Donor Study* reported that some corneas remained clear although the ECD dropped below 500 cells/mm^2^.

Recently, several in vitro and in vivo studies provided evidence for the role of the aqueous humor on endothelial cell survival: Total antioxidant capacity and ascorbic acid levels are decreased in eyes with lower endothelial cell densities [33]. Furthermore, several cytokines, for example, interleukin-6, are elevated preoperatively in the aqueous humor in eyes with subsequent graft failure after keratoplasty [34, 35]. In FECD eyes, an upregulation of several genes (e.g. N-cadherin, alpha-SMA, etc.) has been shown in endothelial cells adjacent to guttae leading to an altered microenvironment on endothelial level [36]. These findings might be an interesting therapeutical approach to influence endothelial cell survival.

The loss of endothelial cells we found in our first cohort study about the 5-year results after DMEK was stable during the 5-year period after an initial relatively high loss by 42% at the 1-year visit. The current study, which comprises the data of a subgroup of the patients of the first study with a longer follow-up, showed a further decline after the first five years with a distinct decrease after 6–7 years. This might be attributed to the change in our endothelial cell measurement devices in 2016, which is a major shortage of this study limiting the validity of our ECD results. Unfortunately, internal comparison and validation of the devices are not possible since the older one is not available anymore. To our knowledge, there exist no studies comparing the variability of measurements taken by these two devices.

Another major limitation of this study is the relatively small number of patients fulfilling the required follow-up time of 8–10 years.

The retrospective setting of the study contributed to the high drop-out rate of the study. Patients undergoing DMEK at our Department visit the out-patient clinic after 1 month, 3 months, and annually after the first year. A reason for the high loss of patients for the long-term controls is attributed to the fact, that in the years between 2009 and 2011 only few sites in Europe offered the innovation of DMEK surgery. Therefore, most patients traveled a long distance for the surgery and were not willing or able to attend the annual follow-up visits at our clinic. The mean age of patients at time of surgery was 63 ± 9 years, i.e. the average age was 73 years at the 10-year follow-up, making annual visits more troublesome.

Another limitation of this study is the lack of eyes with pseudophakic bullous keratopathy (PBK). This is caused mainly by the small number of patients included and by the lower rate of PBK compared to FECD in eyes undergoing endothelial keratoplasty in our department. Further studies addressing the long-term outcome of DMEK in PBK are necessary.

Since the surgical technique has been standardized by several modifications, complications—especially graft detachments—have become less frequent in the last years [38]. All eyes included in this study underwent surgery in the early period of DMEK evolution. Thereby, the relatively rebubbling rate in this study cohort can be explained. One might expect a superior graft survival and long-term stability of DMEK in the future due to the standardized surgical technique. However, in two comparative studies of eyes in which DMEK surgery was performed in different stages of the learning curve of corneal surgeons, no significant difference of the ECD loss between less and more experienced surgeons was found during the follow-up period of 6 and 12 months, respectively, even though the rate of graft detachments dropped dramatically during the study period because of further technical improvements. [37, 38].

Further studies about the long-term course after DMEK are desired since it is unknown if DMEK grafts are going to fail after 10–15 years when the endothelial cell count crosses the border of corneal compensatory capability. In addition, the long-term prognosis of eyes with difficult preoperative situations (for example prior glaucoma surgery) or in eyes with indications other than FECD should be analyzed in the future.

The results of this evaluation of the 10-year outcomes in a small subset of DMEK grafts show the natural development of these eyes with stable visual function over 10 years, despite a clear trend of endothelial cell loss over time. The value of this study is curtailed by the high loss to follow-up in the original DMEK cohort. Prospective longitudinal cohort studies are needed to determine the long-term survival of DMEK grafts.

## Supplementary Information

Below is the link to the electronic supplementary material.

Supplementary file1 (EPS 22 KB)

Supplementary file2 (DOCX 30 KB)

Supplementary file3 (DOCX 15 KB)

## Data Availability

All authors state that data and materials comply with field standards.

## Abbreviations

BCVA: Best corrected visual acuity
CCT: Central corneal thickness
DMEK: Descemet membrane endothelial keratoplasty
DSAEK: Descemet’s stripping automated endothelial keratoplasty
ECD: Endothelial cell density
FECD: Fuchs’ endothelial corneal dystrophy
logMAR: Logarithm of the minimum angle of resolution
PK: Penetrating keratoplasty
SD: Standard deviation
