# Supplementary material for: Descemet membrane endothelial keratoplasty: analysis of clinical outcomes of patients with 8–10 years follow-up
Source: Int Ophthalmol. 2022 Jan 8;42(6):1789–98. doi: 10.1007/s10792-021-02176-3 (PMC9156484; doi:10.1007/s10792-021-02176-3)
Supplement: Supplementary file 2 — Supplementary file2 (DOCX 30 KB) [file 10792_2021_2176_MOESM2_ESM.docx]

**Supplemental table 1 - Cumulative graft survival probability (Kaplan-Meier estimator)**

The medical files of all DMEK surgeries (n=450) performed between July 2009 and June 2012 were analyzed regarding the follow-up time. The follow-up time of all eyes undergoing surgery during this interval was obtained and the graft survival rate was calculated.

| **Follow-up (months)** | **Remaining cases** | **Number of events** | **Cumulative survival probability**  **± standard error** | **Lower 95% confidence interval** | **Upper 95% confidence interval** |
| --- | --- | --- | --- | --- | --- |
| 0 | 438 | 0 | 1.00 | 1.00 | 1.00 |
| 3 | 411 | 6 | 0.99 ± 0.01 | 0.95 | 0.99 |
| 6 | 394 | 6 | 0.97 ± 0.01 | 0.92 | 0.99 |
| 9 | 355 | 3 | 0.96 ± 0.01 | 0.90 | 0.99 |
| 12 | 344 | 1 | 0.96 ± 0.01 | 0.89 | 0.98 |
| 24 | 288 | 3 | 0.95 ± 0.01 | 0.87 | 0.98 |
| 36 | 221 | 3 | 0.94 ± 0.01 | 0.84 | 0.98 |
| 48 | 169 | 1 | 0.93 ± 0.01 | 0.83 | 0.98 |
| 60 | 142 | 3 | 0.91 ± 0.02 | 0.78 | 0.97 |
| 72 | 123 | 2 | 0.90 ± 0.02 | 0.75 | 0.97 |
| 84 | 98 | 1 | 0.89 ± 0.02 | 0.72 | 0.96 |
| 96 | 68 | 1 | 0.87 ± 0.03 | 0.69 | 0.96 |
| 108 | 39 | 2 | 0.83 ± 0.04 | 0.58 | 0.94 |
| 120 | 21 | 1 | 0.80 ± 0.05 | 0.46 | 0.92 |
