# Supplementary material for: Descemet membrane endothelial keratoplasty: analysis of clinical outcomes of patients with 8–10 years follow-up
Source: Int Ophthalmol. 2022 Jan 8;42(6):1789–98. doi: 10.1007/s10792-021-02176-3 (PMC9156484; doi:10.1007/s10792-021-02176-3)
Supplement: Supplementary file 3 — Supplementary file3 (DOCX 15 KB) [file 10792_2021_2176_MOESM3_ESM.docx]

**Supplemental table 2**

Analysis of best corrected visual acuity, endothelial cell density, and central corneal thickness in eyes with a follow-up of ten years only (n=21).

Wilcoxon signed-rank test was used for comparisons of the mean value with the results at the previous follow-up visit. (BCVA = best-corrected visual acuity; SD = standard deviation; logMAR = logarithm of the minimum angle of resolution, y = years)

1. Best corrected visual acuity (only eyes without ocular comorbidities)

|  | Base-line | 1 y | 2 y | 3 y | 4 y | 5 y | 6 y | 7 y | 8 y | 9 y | 10 y |
| --- | --- | --- | --- | --- | --- | --- | --- | --- | --- | --- | --- |
| Mean ± SD  (logMAR) | 0.62  ± 0.42 | 0.17  ± 0.11 | 0.13  ± 0.10 | 0.14  ± 0.11 | 0.13  ± 0.08 | 0.14  ± 0.09 | 0.12  ± 0.12 | 0.13  ± 0.10 | 0.11  ± 0.12 | 0.15  ± 0.10 | 0.10  ± 0.13 |
| n | 19 | 16 | 16 | 16 | 13 | 9 | 12 | 12 | 13 | 16 | 19 |
| P value | - | **0.001** | 0.671 | 0.722 | 0.483 | 0.892 | 0.785 | 0.752 | 0.684 | 0.752 | 0.128 |

1. Endothelial cell density

|  | Base-line | 1 month | 3 months | 1 y | 2 y | 3 y | 4 y | 5 y | 6 y | 7 y | 8 y | 9 y | 10 y |
| --- | --- | --- | --- | --- | --- | --- | --- | --- | --- | --- | --- | --- | --- |
| Mean  ± SD  (cells/mm²) | 2643  ± 2542 | 1531  ± 171 | 1407  ± 311 | 1471  ± 226 | 1303  ± 271 | 1354  ± 240 | 1345  ± 314 | 1496  ± 198 | 1224  ± 295 | 729  ± 169 | 709  ± 121 | 724  ± 176 | 728  ± 167 |
| Median | 2640 | 1550 | 1347 | 1414 | 1240 | 1419 | 1324 | 1500 | 1240 | 669 | 690 | 696 | 694 |
| N | 21 | 14 | 17 | 18 | 16 | 15 | 10 | 11 | 9 | 10 | 14 | 15 | 21 |
| P value | - | **0.001** | **0.041** | 0.233 | 0.132 | 0.650 | 0.575 | 0.686 | **0.042** | 0.116 | 0.484 | 0.767 | 0.480 |

1. Central corneal thickness

|  | Base-line | 1 y | 2 y | 3 y | 4 y | 5 y | 6 y | 7 y | 8 y | 9 y | 10 y |
| --- | --- | --- | --- | --- | --- | --- | --- | --- | --- | --- | --- |
| Mean  ± SD  (μm) | 663  ± 64 | 528  ± 32 | 539  ± 37 | 551  ± 46 | 558  ± 35 | 574  ± 36 | 559  ± 32 | 566  ± 46 | 579  ± 59 | 567  ± 37 | 569  ± 41 |
| Median | 656 | 525 | 531 | 538 | 547 | 570 | 556 | 555 | 564 | 566 | 563 |
| n | 21 | 19 | 16 | 17 | 13 | 12 | 12 | 12 | 15 | 17 | 21 |
| P value | - | **<0.001** | **0.003** | 0.551 | 0.064 | 0.092 | 0.086 | 0.333 | 0.248 | 0.563 | 0.698 |
